# Sera of Neuromyelitis Optica Patients Increase BID-Mediated Apoptosis in Astrocytes

**DOI:** 10.3390/ijms23137117

**Published:** 2022-06-27

**Authors:** Omri Zveik, Ariel Rechtman, Nitzan Haham, Irit Adini, Tamar Canello, Iris Lavon, Livnat Brill, Adi Vaknin-Dembinsky

**Affiliations:** 1 Department of Neurology and Laboratory of Neuroimmunology, The Agnes-Ginges Center for Neurogenetics, Hadassah-Hebrew University Medical Center, Jerusalem 91120, Israel; omrizv@gmail.com (O.Z.); arielrechtman@gmail.com (A.R.); nitzan.haham@mail.huji.ac.il (N.H.); tamarcanello@gmail.com (T.C.); irisl@hadassah.org.il (I.L.); livnatb1@gmail.com (L.B.); 2 Faculty of Medicine, Hebrew University of Jerusalem, Jerusalem 91120, Israel; 3 Department of Surgery, Harvard Medical School, Center for Engineering in Medicine & Surgery, Massachusetts General Hospital, 51 Blossom Street, Boston, MA 02114, USA; iadini@mgh.harvard.edu; 4 Leslie and Michael Gaffin Center for Neuro-Oncology, Hadassah-Hebrew University Medical Center, Jerusalem 91120, Israel

**Keywords:** NMO, astrocytes, apoptosis, BID, inflammation, neuroimmunology

## Abstract

Neuromyelitis optica (NMO) is a rare disease usually presenting with bilateral or unilateral optic neuritis with simultaneous or sequential transverse myelitis. Autoantibodies directed against aquaporin-4 (AQP4-IgG) are found in most patients. They are believed to cross the blood–brain barrier, target astrocytes, activate complement, and eventually lead to astrocyte destruction, demyelination, and axonal damage. However, it is still not clear what the primary pathological event is. We hypothesize that the interaction of AQP4-IgG and astrocytes leads to DNA damage and apoptosis. We studied the effect of sera from seropositive NMO patients and healthy controls (HCs) on astrocytes’ immune gene expression and viability. We found that sera from seropositive NMO patients led to higher expression of apoptosis-related genes, including BH3-interacting domain death agonist (BID), which is the most significant differentiating gene (*p* < 0.0001), and triggered more apoptosis in astrocytes compared to sera from HCs. Furthermore, NMO sera increased DNA damage and led to a higher expression of immunological genes that interact with BID (TLR4 and NOD-1). Our findings suggest that sera of seropositive NMO patients might cause astrocytic DNA damage and apoptosis. It may be one of the mechanisms implicated in the primary pathological event in NMO and provide new avenues for therapeutic intervention.

## 1. Introduction

Acute optic neuritis (ON) is the most common optic neuropathy affecting young adults [1]. It is characterized by reduced visual acuity, color desaturation, scotoma, and may induce ocular pain [2]. ON is present at onset in more than 50% of patients with Neuromyelitis optica (NMO) [3,4], a rare autoimmune inflammatory demyelinating syndrome of the central nervous system (CNS) [3]. The detection of antibodies against the astrocytic water channel aquaporin-4 (AQP4-IgG) distinguished NMO from other demyelinating disorders and re-defined NMO as an antibody-mediated autoimmune disease [5,6,7,8].

In the brain, AQP4 is mainly concentrated in astrocyte end-feet at pial and ependymal surfaces in contact with the cerebrospinal fluid (CSF) and blood vessels [9]. Astrocytes are critically important in the formation and maintenance of the blood–brain barrier (BBB), in maintaining ion and water homeostasis, neurotransmitter recycling, formation and maintenance, as well as the regulation of neural synaptogenesis [10,11,12,13,14,15]. They are known for their roles in immune response as well, including: the expression of immune-related receptors [16], synthesis of the complement components, and production of both immunomodulatory and immunopathogenic cytokines and chemokines [17,18,19].

AQP4-IgG primarily targets astrocytes in the CNS, resulting in secondary demyelination [20,21,22], which frequently leads to severe neurological deficits, including blindness and paraplegia [23,24]. It is thought that AQP4-IgG enters the CNS through areas of increased BBB permeability and binds selectively to AQP4 on astrocytes. The binding of the autoantigen is followed by complement activation and astrocyte destruction, leading to massive infiltration of leukocytes, particularly T and B lymphocytes, eosinophils, and neutrophils [25,26,27]. Although the role of autoantibodies and B cells remains the key factor in NMO pathogenesis, the primary pathological event remains elusive [21,26,27,28].

Here, we examined the effect of sera from seropositive NMO patients and healthy controls (HCs) on astrocytes’ immune gene expression and viability. The ability to identify the pathological changes astrocytes undergo upon exposure to sera of NMO patients is of interest, as this may lead to the development of novel therapies in NMO patients.

## 2. Results

### 2.1. Immunological Gene Expression Profiling of Human Astrocytes Cultured with NMO Sera

In order to study the effect of sera of seropositive NMO patients on astrocytes, we performed a large-scale gene expression array of 580 immune-related genes using the NanoString nCounter code set panel. Human astrocytes were cultured with human sera (10% of media) of either seropositive NMO patients or HCs for 24 h (*n* = 4 for each group).

**Figure 1:**
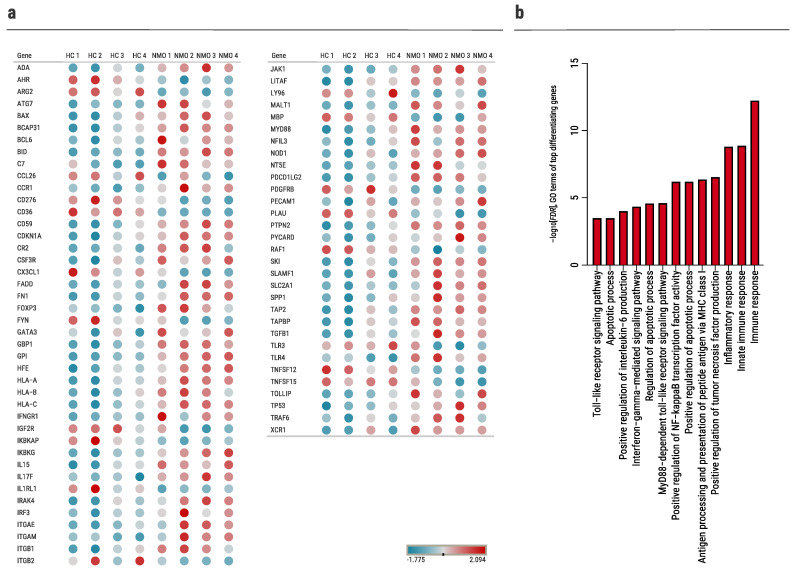
Immunological gene expression profiling in human primary astrocytes cultured with NMO sera. NanoString nCounter analysis of astrocytes cultured with human sera from HCs (*n* = 4) and NMO (*n* = 4) patients. (**a**) Dot plot illustrating top 73 differentially expressed genes: blue denotes low expression, red denotes high expression (padj < 0.1), (**b**) analysis of top 73 differentially expressed genes in NMO cultured astrocytes versus HC cultured astrocytes. Plot of the top enriched gene ontology (GO) terms (focus on ‘function’ in GOrilla), sorted by –log10[FDR].

Out of 580 genes, we identified 73 genes that differentiate significantly (padj < 0.1) between the two groups (NMO vs. HCs, Figure 1a and Table S1). Functional and enrichment analyses of the differently expressed genes were performed using DAVID [29]. Top significant Gene Ontology (GO) terms related to the apoptotic process, Toll-like receptor (TLR) signaling pathway, regulation of Interleukin (IL)-6 production, and antigen processing and presentation (Figure 1b). In addition, among the 73 significantly differentiating genes are complement-related genes such as CR2 and C7, which are known to be involved in NMO pathogenesis [30].

### 2.2. BID Pathway in Neuromyelitis Optica

#### 2.2.1. Increased *BID* Expression in Astrocytes Following Exposure to Sera of Seropositive NMO Patients

**Figure 2:**
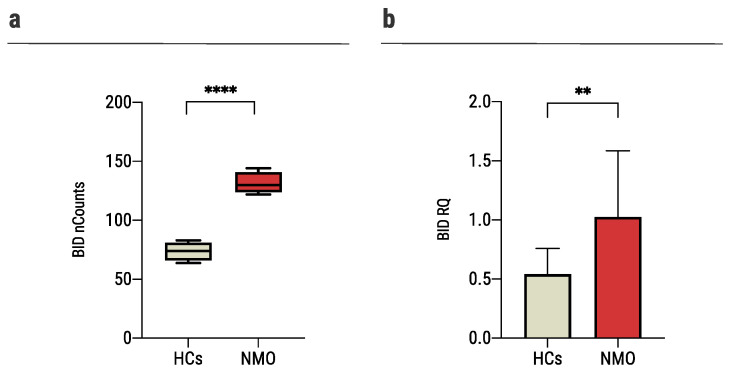
Exposure of astrocytes to sera of NMO patients increased *BID* expression. (**a**) The expression of *BID* increased significantly in human astrocytes cultured with sera of NMO patients (131.4 ± 9.3, *n* = 4) compared to astrocytes cultured with sera of HCs (73.7 ± 8.0, *n* = 4), as was determined with NanoString nCounter technology, (**b**) The expression of *BID* increased significantly in mouse astrocytes cultured with sera of NMO patients (1.02 ± 0.6, *n* = 18) compared to the HCs (0.54 ± 0.22, *n* = 15), as was determined using rt-QPCR. Each assay was repeated independently at least three times. Significance was determined by unpaired two-tailed student’s *t*-test (*p* ** ≤ 0.01, **** ≤ 0.0001). Error bars in all graphs represent standard deviation.

BH3-interacting domain death agonist (*BID*) is the most significant differentiating gene between the two groups as found in the nCounter analysis (NMO: 131.4 ± 9.3 nCounts, HCs: 73.7 ± 8.0 nCounts, *p* < 0.0001, Figure 2a). In a validation experiment performed on primary astrocytes of mice and a larger group of patients (NMO: *n* *=* 18, HCs: *n* = 15) using rt-QPCR analysis, we confirmed that the expression level of *BID* was significantly increased in astrocytes cultured with sera of seropositive NMO patients compared to HCs (1.02 ± 0.6 RQ and 0.54 ± 0.22 RQ, *p* = 0.0037, Figure 2b). 

*BID* is an essential member of the apoptotic process [31]. Also, it is involved in the regulation of DNA damage [32], and the regulation of innate immunity and inflammation via TLR and nucleotide-binding oligomerization domain containing (NOD)-1 signaling and IL-6 production [33,34]. Thus, we next analyzed these pathways in astrocytes cultured with sera obtained from patients with NMO.

#### 2.2.2. Sera of NMO Patients Increase DNA Damage Response in Astrocytes

**Figure 3:**
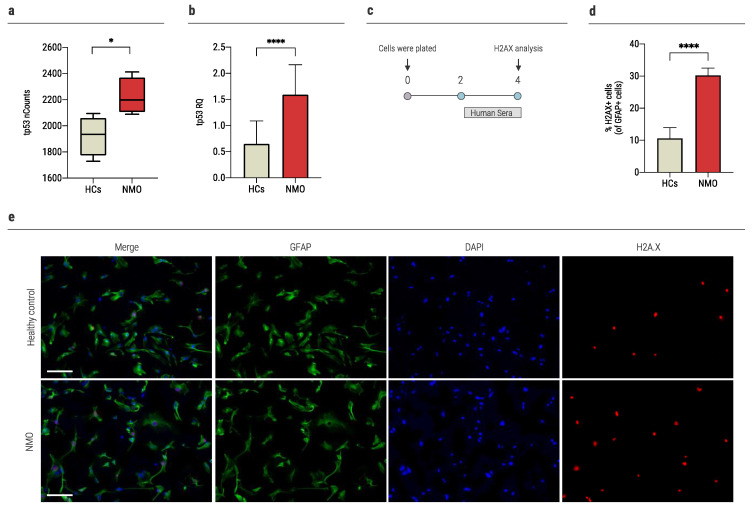
Sera of AQP4+NMO patients increase DNA damage response in astrocytes. (**a**) The expression of *tp53* increased significantly in the human astrocytes cultured with sera of NMO patients (2224.3 ± 139.3, *n* = 4) compared to the HCs (1923.2 ± 150.9, *n* = 4), as was determined with NanoString nCounter technology, (**b**) the expression of *tp53* increased significantly in the astrocytes cultured with sera of NMO patients (1.6 ± 0.6, *n* = 15) vs. 0.64 ± 0.4 (*n* = 12) of the HCs, as was determined using rt-QPCR, (**c**) time-course experiments of H2AX expression. Mouse primary astrocytes were cultured with human sera (20% of media) for 48 h, followed by evaluation of nuclear H2AX expression using immunofluorescence staining, (**d**) nuclear expression of H2AX was determined after culture of 48 h: HCs: 10.6 ± 3.4%; NMO: 30.3 ± 2.2%. Data are means ± SD (*n* = 9 for each group), (**e**) representative immunofluorescence analysis of primary astrocytes cultured with sera from seropositive NMO patient or HC for 48 h (scale bar = 60 μm). Each assay was repeated independently at least three times. Significance was determined by unpaired two-tailed student’s *t*-test (*p* * ≤ 0.05, **** ≤ 0.0001,). Error bars in all graphs represent standard deviation.

Tumor protein P53 (tp53) is an essential regulator of DNA damage, cell-cycle arrest, and the apoptotic process. It is known to regulate important genes that may initiate the intrinsic apoptotic pathway, such as *BID* [35,36]. In the nCounter analysis, *tp53* was significantly upregulated in NMO compared to HCs (2224.3 *±* 139.3 nCounts and 1923.2 *±* 150.9 nCounts, *p* = 0.026, Figure 3a). In a validation experiment performed using rt-QPCR, we found that the expression level of *tp53* is significantly increased in astrocytes cultured with seropositive NMO sera compared to sera of HCs (1.6 *±* 0.6 RQ and 0.64 *±* 0.4 RQ, respectively, *p* < 0.0001, Figure 3b).

To address DNA damage response, we examined histone family member X (H2AX) expression in primary astrocytes of mice following exposure to sera of AQP4+ patients. Immunofluorescence staining was performed following exposure to human sera (20% of media) for 48 h (Figure 3c). Exposure of astrocytes to sera from seropositive NMO patients resulted in significantly higher percentages of H2AX-expressing cells compared with the exposure to sera of HCs and no sera (30.3 *±* 2.2%, 10.6 *±* 3.4%, and 1.7 *±* 0.6%, *p* < 0.0001, Figure 3d,e).

#### 2.2.3. Increased Apoptosis of Astrocytes Following Exposure to NMO Sera

**Figure 4:**
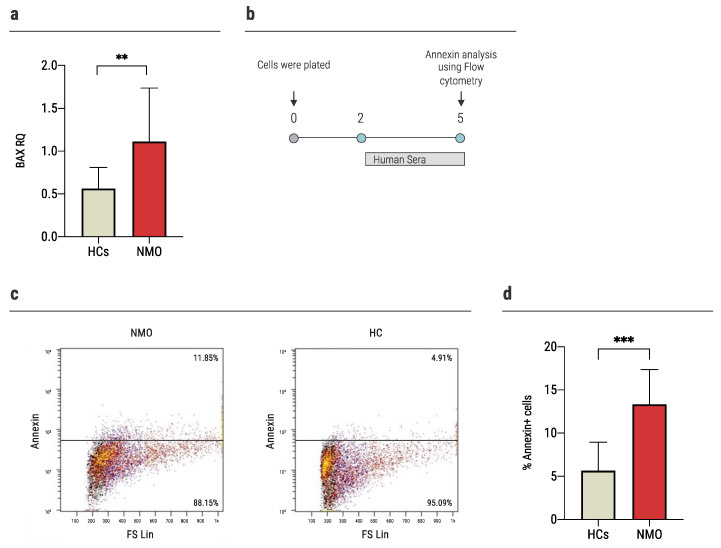
Exposure of NMO sera increased apoptosis in astrocytes. (**a**) The expression of *BAX* increased significantly in the astrocytes cultured with sera of NMO patients (1.11 ± 0.62, *n* = 17) vs. 0.56 ± 0.24 (*n* = 13) of the HCs, as was determined using rt-QPCR, (**b**) time-course experiments of annexin levels. Primary astrocytes were cultured with human sera (20% of media) for 72 h, followed by evaluation of apoptosis expression using flow cytometry, (**c**) representative flow cytometry analysis of apoptotic astrocytes upon culture with sera obtained from seropositive NMO patients or HCs, (**d**) higher levels of apoptosis among astrocytes cultured with sera of NMO patients (13.34 ± 4.03%, *n* = 10), compared to HCs (5.7 ± 3.3%, *n* = 10), as evaluated using annexin staining. Each assay was repeated independently at least three times. Significance was determined by unpaired two-tailed student’s *t*-test (*p* ** ≤ 0.01, *** ≤ 0.001). Error bars in all graphs represent standard deviation.

In response to pro-apoptotic signaling, BID interacts with other Bcl-2 family proteins, such as BCL2 Associated X (BAX), to initiate the apoptotic process [31]. *BAX* was found to be significantly upregulated in astrocytes cultured with NMO sera compared to HCs in the nCounter analysis (7680.1 ± 449.7 nCounts and 6270.4 ± 712.7 nCounts, *p =* 0.038). This was established in a validation experiment as described above (NMO: 1.11 ± 0.62 RQ, HCs: 0.56 ± 0.24 RQ, *p =* 0.0059, Figure 4a).

In order to assess the apoptosis level, we performed annexin staining of mouse primary astrocytes following exposure to sera of AQP4+ NMO patients or HCs. Astrocytes were cultured with human sera (20% of media) for 72 h. Then, we evaluated the astrocytes’ apoptosis using flow cytometry (Figure 4b). Exposure of astrocytes to sera of NMO patients resulted in significantly higher annexin staining compared to the exposure to sera of HCs (13.34 ± 4.03% vs. 5.7 ± 3.3%, *p* = 0.0002, Figure 4c,d).

#### 2.2.4. Volumetric Brain Loss Correlates with *BID* and Annexin Levels of Astrocytes Cultured with Sera of NMO Patient

We analyzed the volume of 14 different brain structures using the Volbrain platform. We assessed the correlation of each patient’s volumetric data and the effect of the same patient’s sera on mouse astrocytes, as measured by *BID* expression and annexin levels. 

**Figure 5:**
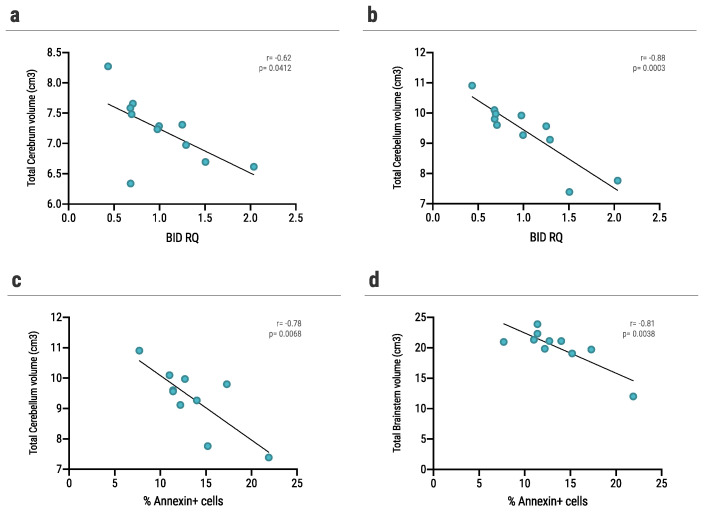
Correlation between *BID* and annexin levels in sera-cultured astrocytes and brain volume. (**a**,**b**) Correlation between *BID* RQ expression levels of mouse astrocytes following culture with sera of AQP4+ patients and (**a**) total cerebrum volume (r = −0.62, *p* = 0.0412), (**b**) total cerebellum volume (r = −0.88, *p =* 0.0003), (**c**,**d**) correlation between annexin levels of mouse astrocytes following culture with sera of AQP4+ patients and (**c**) total cerebellum volume (r = −0.78, *p* = 0.0068), (**d**) total brainstem volume (=−0.81, *p =* 0.0038). Correlation was determined by Pearson correlation test. Differences were considered significant at *p* < 0.05.

We found a significant negative correlation between *BID* expression levels of mouse astrocytes cultured with sera of NMO patient and total cerebrum (r = −0.62, *p* = 0.0412, Figure 5a) and cerebellum volume (r = −0.88, *p* = 0.0003, Figure 5b) of the same patient. Furthermore, we assessed the correlation between brain volume and annexin levels of mouse astrocytes cultured with sera of NMO patient. We found a significant negative correlation between annexin levels and cerebellum volume (r = −0.78, *p* = 0.0068, Figure 5c) and brainstem volume (r = −0.81, *p* = 0.0038, Figure 5d) of the same patient.

### 2.3. Increased Pro-Inflammatory Gene Expression upon Exposure of Astrocytes to Sera of NMO Patients

To better understand the immune effect of NMO sera on astrocytes, we validated two representative genes that were found to differ significantly between the groups. 

**Figure 6:**
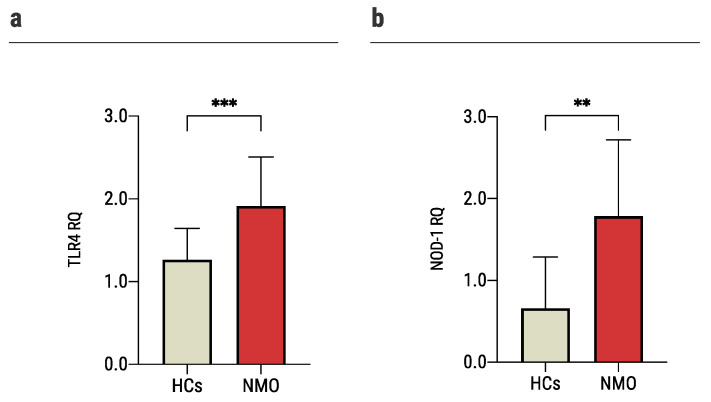
Increased pro-inflammatory gene expression upon exposure of astrocytes to sera of NMO patients. Quantitative PCR gene expression analysis of: (**a**) the expression of *TLR4* increased significantly in astrocytes cultured with sera of NMO patients (1.9 ± 0.6, *n* = 19) vs. 1.26 ± 0.4 (*n* = 19) of astrocytes cultured with sera of HCs, (**b**) the expression of *NOD-1* increased significantly in astrocytes cultured with sera of NMO patients (1.8 ± 0.9, *n* = 15) vs. 0.66 ± 0.62 (*n* = 11) of astrocytes cultured with sera of HCs. Each assay was repeated independently at least three times. Significance was determined by unpaired two-tailed student’s *t*-test (*p* ** ≤ 0.01, *** ≤ 0.001). Error bars in all graphs represent standard deviation.

One of the major signaling pathways was TLR signaling pathway (Figure 1b). TLR4 is known for its roles in pathogen recognition and activation of innate immunity [37], but also for its involvement in autoimmune disorders such as multiple sclerosis [38]. Additionally, TLR4 interacts with BID both in innate immune pathways [33] and the apoptotic pathway [39]. In nCounter analysis, the expression of *TLR4* was significantly higher among astrocytes exposed to sera of seropositive NMO patients (1597.1 ± 146.7 nCounts) compared to sera of HCs (1218.9 ± 136.3 nCounts). These findings were further validated on mouse primary astrocytes and a larger group of patients using rt-QPCR analysis (NMO: 1.9 ± 0.6 RQ, HCs: 1.26 ± 0.4 RQ, *p* = 0.0003, Figure 6a).

NOD-1 plays a key role in innate immune [40] and activated TLR4 signaling [41] pathways. It is also regulated by BID, and their interactions may eventually lead to NF-kB activation [33]. BID is required to activate host defense mechanisms to control bacterial infections but may also exacerbate immune-mediated inflammatory disease [33]. In nCounter analysis, *NOD-1* expression levels were significantly higher among astrocytes exposed to NMO sera compared to HCs (188.11 ± 17.01 nCounts, and 127.4 ± 28.0 nCounts, respectively, *p* = 0.009). These findings were further validated as described above (NMO: 1.8 ± 0.9 RQ, HCs: 0.66 ± 0.62 RQ, *p* = 0.002, Figure 6b).

### 2.4. Sera of NMO Patients Stimulates a Repair Process

Following our observation that sera of seropositive NMO patients increased the expression of both genes involved in apoptosis and the TLR signaling pathway, we explored two immunological genes involved in synaptogenesis and known to reduce neuronal damage. 

**Figure 7:**
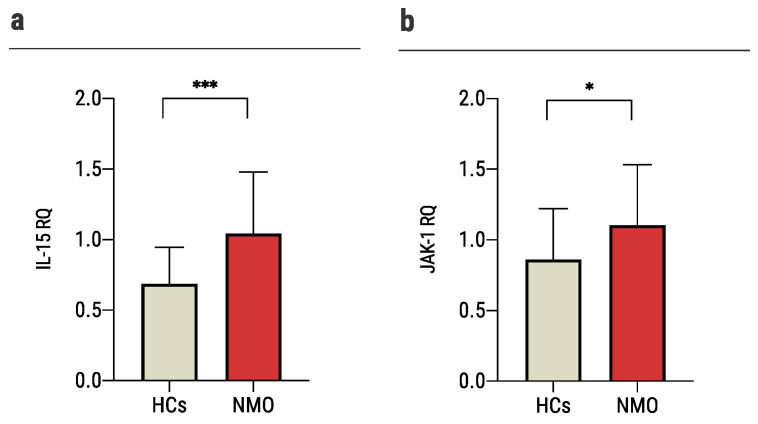
Sera of NMO patients stimulates repair process. Quantitative PCR gene expression analysis of: (**a**) the expression of *IL-15* increased significantly in astrocytes cultured with sera of NMO patients (1.1 ± 0.5, *n* = 31) vs. 0.72 ± 0.3 (*n* = 24) of astrocytes cultured with sera of HCs, (**b**) the expression of *JAK-1* increased significantly in astrocytes cultured with sera of NMO patients (1.1 ± 0.4, *n* = 32) vs. 0.86 ± 0.4 (*n* = 24) of astrocytes cultured with sera of HCs. Each assay was repeated independently at least three times. Significance was determined by unpaired two-tailed student’s *t*-test (*p* * ≤ 0.05, *** ≤ 0.001). Error bars in all graphs represent standard deviation.

IL-15 is known to be upregulated in the CNS after injury [42,43,44,45,46]. Its expression is known to be related to NMO progression: high expression is implicated in reduced lesion size, attenuation of BBB leakage and tight junctions lost, reduced brain infiltration of immune cell subsets, and promotion of astrocytes survival [47]. IL-15 induces the activation of JAK1. Studies suggested that this cytokine may increase the expression of apoptosis inhibitor BCL2L1/BCL-x(L) [48]. Both *IL-15* and *JAK-1* were significantly upregulated among astrocytes exposed to NMO sera compared to HCs (*IL-15*: 185.8 ± 13.4 nCounts vs. 137.6 ± 24.7 nCounts; *JAK-1*: 4356.9 ± 175.1 nCounts vs. 3818.1 ± 130.02 nCounts). In validation experiments as described above, we found that the expression levels of *IL-15* and *JAK-1* were significantly increased in astrocytes cultured with NMO sera compared to HCs (*IL-15*: 1.1 ± 0.5 RQ vs. 0.72 ± 0.3 RQ, *p* = 0.0008; and *JAK-1*: 1.1 ± 0.4 RQ vs. 0.86 ± 0.4 RQ, *p* = 0.029, Figure 7a,b).

Taken together, these data suggest that exposure of astrocytes to NMO sera triggers not only a damaging cascade but also a repair process, which may eventually serve as a therapeutic target.

## 3. Discussion

In the current study, we found that sera from NMO patients have a differential effect on astrocytes’ immune gene expression. Sera from NMO patients led to increased apoptosis of astrocytes compared to sera from HCs. In addition, it also led to higher expression of DNA damage marker, H2AX, and higher expression of immunological genes, such as *TLR4* and *NOD-1*.

Although NMO has been studied extensively, it is still not entirely clear what the primary pathological event is [21,26,27,28]. One accepted theory is that complement activation is initiated upon binding to the autoantigen, leading to astrocyte destruction and secondary demyelination and axonal damage [25,26,27]. It is in line with our result of higher expression of complement-related genes such as CR2 and C7 in human astrocytes cultured with sera of NMO patients. Other studies suggested that astrocyte damage may produce a toxic bystander effect on oligodendrocytes which can lead eventually to demyelination [49,50,51]. An additional possible explanation may be that following the primary event of the interaction of AQP4-IgG and astrocytes, there is an increase in DNA damage, increased expression of *BID*, and a higher level of apoptosis. The increased expression of *BID* also leads to higher expression of *TLR4* and *NOD-1*. Understanding the mechanisms leading to NMO pathology may promote the development of new therapeutic interventions.

Another question regarding NMO pathology is why NMO lesions localize to the optic nerve. Previous works suggested that the restricted diffusion of AQP4 and other pro-inflammatory factors in the optic nerve may increase their concentration [52,53]. Also, the unique anatomy of the optic nerve is exceptional in myelinated tracks, which may provide another explanation for ON as well [54]. Furthermore, the high expression of AQP4 on astrocytes in the optic nerve compared to the brain remains the main reason for the susceptibility of the optic nerve in NMO patients [55].

Initially, we chose to focus on the apoptotic process. *BID*, the most significant differentiating gene in the nCounter analysis, is a pro-apoptotic member of the Bcl-2 protein family [31]. We assessed apoptosis in astrocytes exposed to NMO and HCs sera using annexin staining. We found higher levels of apoptotic astrocytes (2.34 time-fold) among the NMO group compared to the HCs. These observations align with a previous work by Brill et al., which showed an increase in apoptosis and *BID* expression levels in peripheral blood mononuclear cells (PBMCs) of NMO patients compared to HCs [56]. Additionally, we found a significant negative correlation between annexin levels or *BID* expression of mouse astrocytes cultured with sera of NMO patients and their volumetric MRI data. Liu et al. observed lower brain volume in NMO patients compared to HCs [57]. Previous works suggested a correlation between brain atrophy and cognitive impairment in NMO patients [58,59]. Our data show that sera of seropositive NMO patients induce more apoptosis of astrocytes in vitro and suggest that a mechanism of programmed astrocytic death by apoptosis might be implicated in the pathology of NMO.

**Figure 8:**
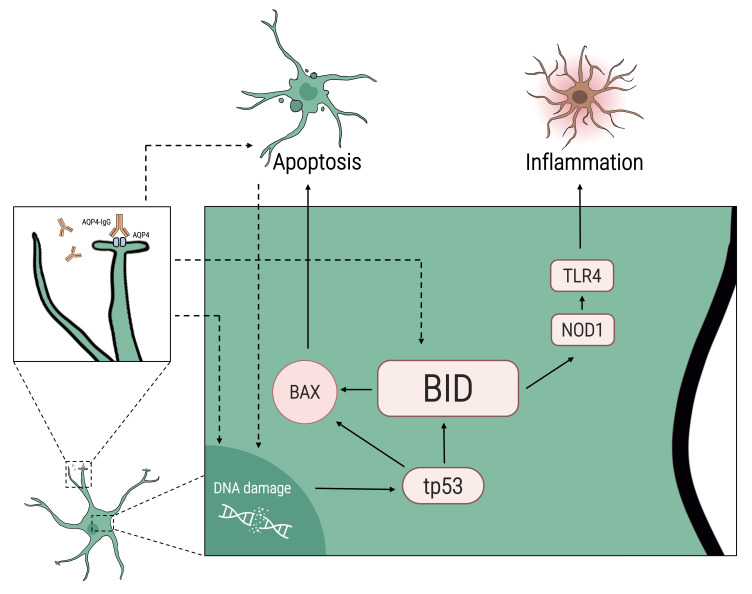
Proposed scheme for BID-mediated apoptosis in astrocytes in NMO. Following culture of astrocytes with sera of NMO patients, we found an increase in the expression of *BID*, *tp53*, *BAX*, *NOD-1*, and *TLR4*. Increased *BID* levels may be linked to NMO pathogenesis through several pathways: The increase in *BID* can mediate the inflammation process in NMO by increasing *NOD-1* and *TLR4*. The interaction of AQP4-IgG and AQP4 receptors on astrocytes leads to complement activation, necrosis/apoptosis, and DNA damage, which in turn may activate cell-cycle regulator *tp53*, leading to BID-mediated apoptosis. Other inflammatory factors in the sera of NMO patients can directly activate the BID pathway or indirectly by increasing DNA damage.

BID is a regulator of the apoptotic process. In response to apoptotic signaling, BID interacts with other Bcl-2 family proteins, such as BAX. We found higher expression of *BAX* in astrocytes cultured with sera of NMO patients compared to HCs. BAX has previously been implicated in mediating nitric oxide-induced apoptosis in astrocytes of the cerebral cortex and the optic nerve through a tp53-dependent pathway [60,61,62]. To initiate apoptosis and activate BAX, BID must be trunked into trunked(t)-BID [63]. It can be due to cues from the extrinsic pathway (such as FADD), the granzyme B pathway, or due to DNA damage response [64]. Our data showed a higher expression of *tp53* in astrocytes cultured with sera of NMO patients compared to HCs. Using H2AX staining, we found that sera of NMO patients led to higher levels of DNA damage in astrocytes compared to sera of HCs. It is possible that one of the signals leading to higher apoptosis in astrocytes cultured with NMO sera is DNA damage (Figure 8). DNA damage leads to activation of cell-cycle regulator tp53, which might lead to BID activation and initiation of the intrinsic pathway.

BID also has a role in inflammation and innate immunity [33]. It is suggested that BID is important for the ability to respond to local or systemic exposure to infection [33]. To do so, BID interacts directly with NOD-1 and activates nuclear factor kappa B (NFκB) and ERK pathways [33]. NOD-1 acts as a pattern-recognition receptor that binds bacterial peptidoglycans and initiates inflammation [65,66]. Previous work has shown that activation of NOD-1 in PBMCs of NMO patients increased IL-6 levels [67]. We found that *NOD-1* is upregulated in astrocytes cultured with NMO sera compared to HCs. Once activated, NOD-1 can interact with TLR4 and is involved in innate immune activation [33,40,41]. Interestingly, three of the leading pathways found in the nCounter analysis are TLR signaling pathway, IL-6 production, and apoptotic process.

Our data reveal that *TLR4* is upregulated in astrocytes cultured with sera of NMO patients at the mRNA expression level. It was previously reported that using bacterial lipopolysaccharide (LPS), a typical TLR4 activator, astrocytes are activated and induce a complex set of molecular reactions mediated by NFκB, mitogen-activated protein kinase (MAPK), and Jak1/Stat1 signaling pathways [68]. This cascade may lead to both pro-inflammatory and anti-inflammatory signals. The use of TLR4 agonist has shown to lead to higher secretion of pro-inflammatory mediators (such as IL-6, IL-17, and IL-1b) and to impede secretion of anti-inflammatory IL-10 in PBMCs of NMO patients [69,70]. Haase et al. showed that macrophages deficient for TLR4 diminished Yersinia-induced apoptosis [39]. They also showed that the extended stimulation of overexpressed TLR4 elicited cellular death in epithelial cells. These suggest the implication of TLR4 not only in the immune response but also in the apoptotic process.

Demyelination and oligodendrocytes loss are two of the most important pathological processes leading to disability in NMO patients [30]. Both pathological events are considered secondary damage to astrocyte dysfunction or inflammatory bystander damage [71]. The crosstalk of astrocytes and oligodendrocytes in the CNS is complex and may lead to different outcomes [72]. Astrocytes can secrete detrimental factors (such as hyaluronan or fibronectin), which may halt remyelination and differentiation of oligodendrocyte progenitor cells into mature myelinating oligodendrocytes [73,74,75,76]. On the other hand, astrocytes play a major role in the homeostatic support of oligodendrocytes and secrete beneficial factors to promote remyelination (such as CXCL12 and IGF-1) [77,78]. We suggest that astrocytic apoptosis may lead to a breach of the homeostatic balance and support of oligodendrocytes, thus, eventually leading to loss of oligodendrocytes and failure of remyelination. Theoretically, it is plausible that following damage and apoptosis, astrocytes are secreting detrimental factors that lead to apoptosis of oligodendrocytes. Moreover, the same BID-mediated apoptosis pathway may occur not only in astrocytes but also in oligodendrocytes of NMO patients. This hypothesis is difficult to assess due to the lack of an animal model for NMO and the technical difficulties in co-culture experiments of both cells.

Upon injury, astrocytes may increase the expression of anti-inflammatory or pro-synaptogenesis genes. We found that *IL-15* is highly expressed in NMO-cultured astrocytes compared to HCs. Its expression is known to be related to NMO progression: high expression is implicated in reduced lesion size, attenuation of BBB leakage and tight junctions lost, reduced brain infiltration of immune cell subsets, and promotion of astrocytes survival [47]. IL-15 induces the activation of JAK1. Studies suggested that this cytokine may increase the expression of apoptosis inhibitor BCL2L1/BCL-x(L) [48]. These data suggest that exposure of astrocytes to NMO sera prompts not only damage cascade, but also a repair process, which may eventually serve as a therapeutic target. Targeting astrocytes with treatments that may induce IL-15 expression may serve as a potential treatment for ON and NMO [79,80].

Further potential therapeutic intervention may be the inhibition of astrocytic apoptosis. Inhibition of cysteine cathepsin B and L in astrocytes was reported to contribute to neuroprotection against cerebral ischemia via blocking the t-BID-mitochondrial apoptotic pathway [81]. Other small molecules such as BI-6C9 or idebenone were previously suggested as inhibitors of BID or BAX-induced apoptosis [61,82]. However, there is a potential risk in inhibiting programmed apoptosis instead of targeting the reason that initiated the process.

The limitations of this study are the relatively small cohort and the use of mouse primary astrocytes. Although, we used both human and mouse cells for the immunological expression data.

In conclusion, we showed that following exposure to sera from seropositive NMO patients, there is an increased expression of both immunological and apoptosis-related genes in astrocytes. Astrocytes undergo more apoptosis and gain DNA damage upon exposure to NMO sera. Our data contribute to the current knowledge regarding astrocytic destruction in NMO pathology, suggesting apoptosis as one of the implicated mechanisms in the primary pathology in NMO. These findings may provide new avenues for therapeutic intervention and furnish a better understanding of disease pathogenesis.

## 4. Materials and Methods

### 4.1. Approvals

The Hadassah Medical Organization Ethics Committee approved this study. All subjects provided written informed consent (0589-08-HMO). The research reported in this study complied with all relevant ethical regulations for animal testing and research and was approved by the Hebrew University Institutional Animal Care and Use Committee (MD-20-16227-1).

### 4.2. Subjects

The patients cohort included 35 NMO patients (26 females, 9 males; mean age at diagnosis 41.9 ± 16.9 years; disease duration 9.4 ± 5.2 years; EDSS 4.47 ± 2.4) and 28 healthy individuals who served as controls (16 females, 10 males; mean age 40.5 ± 15.9 years). All patients were followed at the Neurology clinic in the Neurology Department of Hadassah Medical Center, Jerusalem, Israel. The participants’ characteristics were obtained from medical files from the Neurology clinic. All NMO patients were diagnosed according to 2015 diagnostic criteria [5], and were tested positive to anti-AQP4.

### 4.3. Gene Expression Array and Bioinformatics Analysis

A large-scale gene expression array was performed by utilizing NanoString nCounter technology (NanoString Technologies Inc., Seattle, WA, USA). Total RNA was extracted from human astrocytes (LONZA, Haifa, Israel) cultured with sera of NMO patients and HCs using Tri Reagent BD (Sigma–Aldrich, Rehovot, Israel). Samples were analyzed for 580 immunology genes with the nCounter code set panel (NanoString Technologies Inc., Seattle, WA, USA). The assay is based on direct digital detection of mRNA molecules of interest with the aid of target-specific, color-coded probe pairs, without the use of reverse transcription or amplification. Raw data (following control and reference gene normalization) is analyzed with nsolver analysis software. Following hierarchical clustering, GO pathway enrichment analysis is used to define pathways related to these genes [83]. The Database for Annotation, Visualization and Integrated Discovery (DAVID) [29] was used to study shared biological processes of significant differential genes (https://david.ncifcrf.gov, RRID:SCR_001881, Date: 31 May 2022).

### 4.4. Mouse Primary Astrocytes Culture

Mouse primary astrocytes were isolated from naïve P0 to P1 neonatal *C57/BL6* mice cortices as previously described by Chen et al. [84], with minor modifications [85,86]. Briefly, a mixed glial culture isolated from neonatal mice was grown for 8 days in Dulbecco’s modified Eagle’s medium (DMEM) low glucose (Biological Industries, Kibbutz Beit-Haemek, Israel) supplemented with 5% fetal bovine serum (Biological Industries, Kibbutz Beit-Haemek, Israel), 1 mM sodium pyruvate (Biological Industries, Kibbutz Beit-Haemek, Israel), 1 mM L-glutamine (Sigma–Aldrich, Rehovot, Israel) and 0.6% Gentamycin Sulfate (Biological Industries, Kibbutz Beit-Haemek, Israel). Culture medium was replaced every 2 days. After 8 days, microglia were detached by 30 min shaking at 140 rpm using an orbital shaker. After medium was removal, a new fresh culture medium was added, and OPCs at the top of the astrocyte monolayer were detached by shaking for 18 h at 200 rpm. Media was replaced and astrocytes were grown for further 7 days. Cells were detached from flasks using TrypLE (Thermo Fisher Scientific, Waltham, MA, USA). All cultures expressed high level of GFAP (mean of 96.6% GFAP+ cells, Figure S1).

### 4.5. Apoptosis Assay

Cells were seeded in plates for 24 h. Then, media was replaced for DMEM low glucose (Biological Industries, Kibbutz Beit-Haemek, Israel) supplemented with 1 mM sodium pyruvate (Biological Industries, Kibbutz Beit-Haemek, Israel), 1 mM L-glutamine (Sigma–Aldrich, Rehovot, Israel) and 0.6% Gentamycin Sulfate (Biological Industries, Kibbutz Beit-Haemek, Israel). Human sera of either NMO patients or HCs (20% of media) were added into mouse primary astrocytes media for 72 h. Apoptosis was assessed by Annexin V detection kit (Cat# BG-62700, EMELCA Bioscience, Clinge, The Netherlands). All fluorescence-activated cell sorting (FACS) samples were analyzed in a Beckman coulter FC500 apparatus using the CXP software. Each assay was repeated independently at least three times.

### 4.6. DNA Damage Assay

Cells were seeded in plates for 24 h. Then, media was replaced for DMEM low glucose (Biological Industries, Kibbutz Beit-Haemekm Israel) supplemented with 1 mM sodium pyruvate (Biological Industries, Kibbutz Beit-Haemek, Israel), 1 mM L-glutamine (Sigma–Aldrich, Rehovot, Israel) and 0.6% Gentamycin Sulfate (Biological Industries, Kibbutz Beit-Haemek, Israel). Human sera of either NMO patients or HCs (20% of media) were added into mouse primary astrocytes media for 48 h. Then, DNA damage response was evaluated using anti-H2AX (Cat# sc-517348, Santa Cruz biotechnology Inc., Dallas, TX, USA, 1:100). Each assay was repeated independently at least three times.

### 4.7. RNA Isolation and Reverse Transcription

Mouse primary astrocytes were cultured with 10% human CSF for 24 h. RNA was extracted from cultured astrocytes using Tri-reagent (Sigma–Aldrich, Rehovot, Israel) as previously described [87,88]. The cDNA was synthesized from 250 ng total RNA using the qScript cDNA Synthesis Kit (Quanta Biosciences, Gaithersburg, MD, USA). Quantitative polymerase chain reaction (PCR) was performed using PerfeCTa SYBR Green FastMix Rox (Quanta Biosciences, Gaithersburg, MD, USA). Gene amplification was carried out using the StepOnePlus real-time PCR system (Applied Biosystems, Waltham, MA, USA). The threshold cycle value (2^−ΔCT^) was used for statistical analysis. All target mRNAs were normalized to the Hypoxanthine-guanine phosphoribosyltransferase (HPRT) reference gene. At least three independent experiments were performed; expression of each gene was evaluated in triplicate and is presented as mean mRNA relative quantification ± SD.

Primers used (Agentek, Yakum, Israel):

HPRT F: 5′ CATGGACTGATTATGGACGGAC R: 5′ ACAGAGGGCCACAATGTGATG

BH3-Interacting Domain Death Agonist (BID) F: 5′ GGCTCCTCAGTCCATCTGGTT R: 5′ GCCAGTCACGCACCATCT

Tp53 F: R: F: 5′ GAGGGAGCTCGAGGCTGATAT F: 5′ TTCTCCGAAGACTGGATGACTG

Nucleotide-Binding Oligomerization Domain Containing (NOD)-1 F: 5′ TGAGGAGCAACCTAGGACAAAG R: 5′ CAGCCATAACAGAGATTTGTCTC

Interleukin (IL-)15 F: 5′ AGCCTACAGGAGGCCAAGAA R: 5′ AATGCCCAGGTAAGAGCTTCAA

Janus Kinase 1 (JAK1) F: 5′ GCTCCACTACCGCATGAGGTT R: 5′ TGGAGAATGTCGCCATACAGAC

TLR-4: F: 5′ TGATGACATTCCTTCTTCAACCA R: 5′ TGGTTGAAGAAGGAATGTCATCA

BCL2 Associated X (BAX): F: 5′ AGTGCACAGGGCCTTGAG R: 5′ GCGTGGTTGCCCTCTICT

### 4.8. Immunostaining

For intra-cellular markers, staining was performed on living cells followed by fixation and permeabilization. Anti-Glial fibrillary acidic protein (GFAP; Cat# Z0334, RRID: AB_10013382, Agilent, Santa Clara, CA, USA, 1:50) was used to identify astrocytes, anti-H2AX (Cat# sc-517348, RRID:AB_2783871, Santa Cruz biotechnology Inc., Dallas, TX, USA, 1:100) was used for evaluation of DNA damage response. Goat anti-rabbit Alexa Fluor 488 (Cat# A-11034, RRID: AB_2576217, Invitrogen, Thermo Fisher Scientific, Waltham, MA, USA, 1:200) and goat anti-mouse Alexa Fluor 555 (Cat# A28180, RRID:AB_2536164, Invitrogen, Thermo Fisher Scientific, Waltham, MA, USA, 1:200) were used as secondary antibodies appropriately. Nuclei were counterstained with 4′,6-diamidino-2-phenylindole (DAPI; Cat# H-1200, RRID:AB_2336790, Vector Laboratories, Burlingame, CA, USA). Quantification was performed using ImageJ software (NIH, public domain software) by measuring positively stained cells relative to total DAPI. Quantifications are represented as mean percentages from total DAPI+ cells ± SD and are from at least 15 random fields captured in three or more independent experiments.

### 4.9. MRI Data Acquisition, Processing, and Analysis

Brain MRIs were acquired using demyelination protocol [89]. T1-weighted images were acquired using MRI scanners at Hadassah Ein Kerem medical center as described before [90]. Volumetric data were extracted using volBrain (http://volbrain.upv.es, Date: 1 December 2021) platform. VolBrain software contains advanced pipelines and automatically provides volumetric information of the brain MR images at different scales [91]. Volbrain provide volumes of total brain, total white matter, total gray matter, cerebrum, cerebellum, brainstem, lateral ventricles, caudate, putamen, thalamus, globus pallidus, hippocampus, amygdala, and nucleus accumbens.

### 4.10. Statistical Analyses

Unpaired two-tailed student’s t-test, one-way ANOVA with Tukey’s multiple comparisons post hoc test, Mann–Whitney, and Pearson correlation tests were performed. Specific tests are noted in figure legends with significance level annotations. Values are provided as mean ± SD, or as described for each figure. All error bars represent standard deviation.

## Data Availability

Not applicable.

## Supplementary Materials

The following supporting information can be downloaded at: https://www.mdpi.com/article/10.3390/ijms23137117/s1.
