# Supplementary material for: Sera of Neuromyelitis Optica Patients Increase BID-Mediated Apoptosis in Astrocytes
Source: Int J Mol Sci. 2022 Jun 27;23(13):7117. doi: 10.3390/ijms23137117 (PMC9266359; doi:10.3390/ijms23137117)
Supplement: Supplementary file 1 [file ijms-23-07117-s001.zip › Figure S1.pdf]

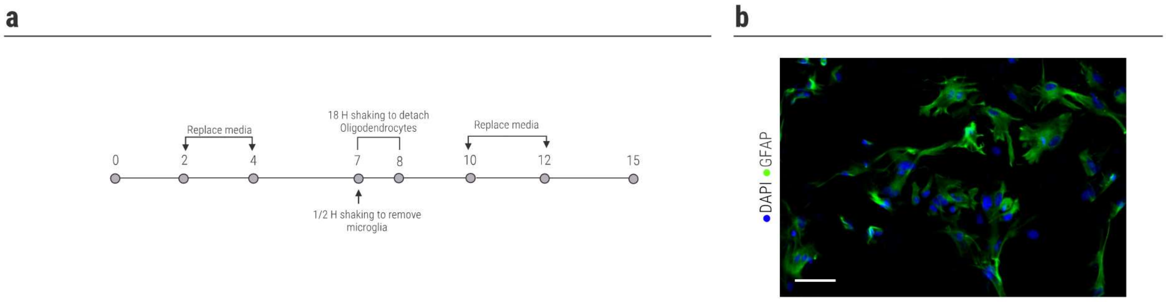

Figure S1. Astrocytes' purity. (**a**) Time course experiments of astrocytes isolation. Astrocytes are isolated from P0 to P1 neonatal mice cortices, cultured in mixed glial culture media and maintained in culture until 7 days in vitro (DIV). Culture media is replaced after 2 DIV and every 2 days thereafter. After 7 DIV, first microglia are detached by 1/2 h shaking and after oligodendrocytes are detached by 18 h shaking. After detachment of microglia and oligodendrocytes, astrocytes are cultured in flasks for 7 days, and culture media is replaced every 2 days, (**b**) Representative immunofluorescence staining of mouse primary astrocytes.
